# Bilateral choroid plexus resection in a 9p hexasomy/tetrasomy mosaic patient

**DOI:** 10.1038/s41439-024-00268-x

**Published:** 2024-02-26

**Authors:** Rei Takada, Takenori Tozawa, Takumi Yamanaka, Masaharu Moroto, Tomoko Iehara, Tomohiro Chiyonobu

**Affiliations:** 1 Department of Pediatrics, Kyoto Prefectural University of Medicine, Kyoto, Japan; 2 Department of Neurosurgery, Kyoto Prefectural University of Medicine, Kyoto, Japan; 3 Department of Pediatrics, Fukuchiyama City Hospital, Kyoto, Japan; 4 Department of Molecular Diagnostics and Therapeutics, Graduate School of Medical Science, Kyoto Prefectural University of Medicine, Kyoto, Japan

**Keywords:** Neurological disorders, Cytogenetics

## Abstract

Previous reports have shown that a gain of the chromosome 9 short arm (9p) is associated with choroid plexus hyperplasia (CPH). Furthermore, CPH can lead to communicating hydrocephalus; however, no cases of CPH with 9p gain requiring choroid plexus resection have been reported. Here, we describe the first case in which a 9p hexasomy/tetrasomy mosaic patient required choroid plexus resection for hydrocephalus. This finding suggested that the 9p copy number is correlated with CPH severity.

Chromosome 9 short arm (9p) gain can occur as trisomy, tetrasomy, or mosaic states, all of which are associated with choroid plexus hyperplasia (CPH), while some cases of communicating hydrocephalus have been reported^1–7^. The exact frequency of 9p gain is unknown; however, more than 200 cases of 9p trisomy, the most common form of 9p gain, have been reported. This trisomy is characterized by growth retardation, intellectual disability, microcephaly, hypertelorism, downslanting palpebral fissures, a wide nasal bridge, a bulbous nose, downturned corners of the mouth, anomalous ears, a short neck, stridor, and short-finger syndrome^3,8^. In comparison, 9p tetrasomy, which is associated with more severe malformations and a worse prognosis^9^, is rarer, with approximately 70 cases reported to date. Furthermore, the complication rate of hydrocephalus is reportedly 45%^1^. Hydrocephalus is defined as the accumulation of cerebrospinal fluid (CSF) in the ventricles of the brain and is caused by an imbalance between CSF production and absorption or obstruction in the ventricles, defined as communication or obstruction, respectively. Hydrocephalus due to excessive CSF production is rare and is usually caused by CPH or choroid plexus tumors. In such cases, large amounts of CSF cannot be absorbed from the abdominal cavity with only a ventriculoperitoneal shunt (VPS), and additional treatment is required^2^.

In the present case, a 10-month-old girl presented to our hospital for hydrocephalus. She was the first child born to healthy, nonconsanguineous Japanese parents with no relevant family history. The baby was born at 40 weeks and 6 days, with a birth weight of 2726 g and an Apgar score of 7/8. Her head circumference was 32 cm (10.3 percentile for gestational age) at birth. She had characteristic facial features (protruding forehead, hypertelorism, a wide nasal bridge, downturned corners of the mouth, ptosis of the corners of the mouth, and anomalous ears); a short neck; patent ductus arteriosus; an atrial septal defect; pulmonary hypertension; right clubfoot; hypoplastic fifth fingernail; and hypoplastic distal phalanges of the first, second, and fifth fingers. Head ultrasound imaging revealed choroid plexus hyperplasia and a cyst (Fig. 1A). Her psychomotor development was delayed: even after 8 months of age, she was unable to follow with her eyes or hold her head up. A G-band was used to investigate the underlying disease, and a mosaic of chromosomes 47,XX and 48,XX from 10 cells was observed, including an extra chromosome that appeared to be derived from chromosome 9 (Supplementary information).

**Fig. 1:**
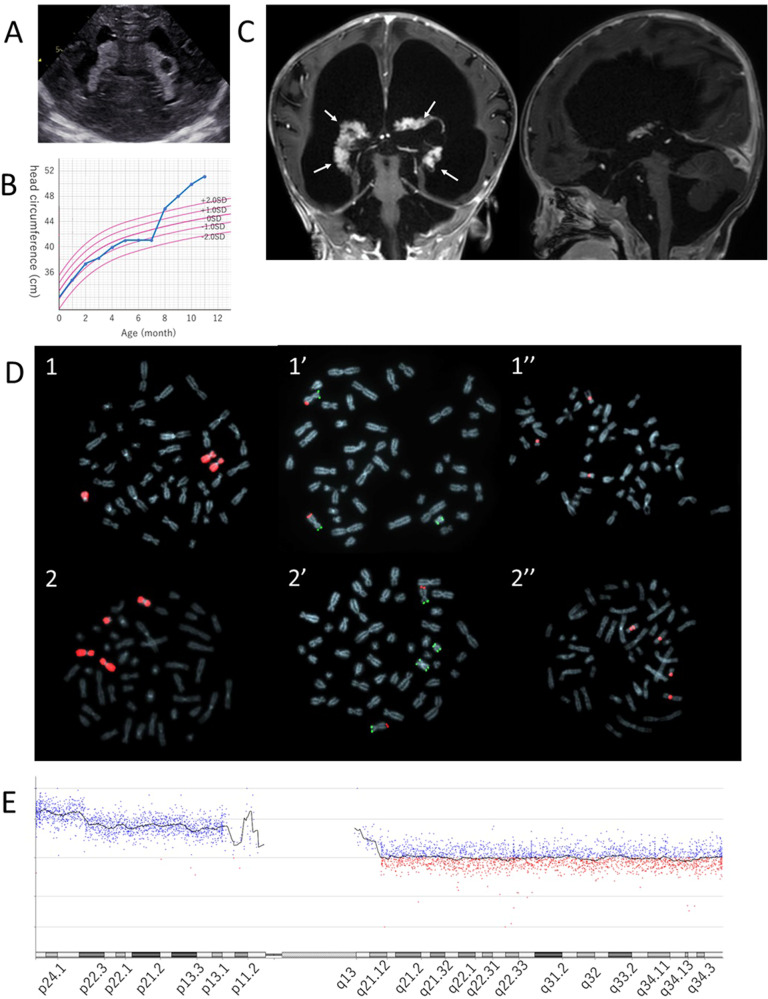
Clinical and genetic information of the patient. **A** Head ultrasound image at taken birth. Choroid plexus hyperplasia and a cyst were observed. **B** Head circumference growth curve. Her head circumference expanded at 8 months of age. **C** Head magnetic resonance imaging at 9 months of age. Hydrocephalus and choroid plexus hyperplasia were observed. **D** Results of FISH (1-1” indicate 47,XX cells, 2-2” indicate 48,XX cells). For 1 and 2, the chromosome 9 painting probe was used. For 1’ and 2’, a subtelomere probe (green, 9p; red, 9q) was used. For 1” and 2”, centromere probes were used. The chromosome 9 painting probes used were as follows: the XCP-kit with Texas Red (Carl Zeiss); chromosome 9 short arm subtelomere probe, Vysis TelVysion 9p Spectrum Green Probe (Vysis); chromosome 9 long arm subtelomere probe, Vysis TelVysion 9q Spectrum Orange Probe (Vysis); and chromosome 9 centromere probe, CEP9 Spectrum Orange Probe (Vysis). **E** Results of the microarray chromosome analysis. A 9p gain was more distal to 9p 24.

Expansion of the head circumference was observed at 8 months of age (Fig. 1B), and head magnetic resonance imaging at 9 months showed enlarged ventricles and choroid plexus hyperplasia with no obvious occlusion, leading to a diagnosis of communicating hydrocephalus associated with CPH (Fig. 1C). No obvious psychomotor regression due to hydrocephalus was observed. The patient continued to experience progressive enlargement of the head circumference and nystagmus and was admitted to the hospital for surgical treatment at 10 months.

Fluorescence in situ hybridization (FISH) and microarray chromosome analysis were performed as additional detailed chromosome examinations. Cells harboring 47,XX (Fig. 1D1–1”) and 48,XX (Fig. 1D2–2”) were closely examined using FISH to determine the origin of the extra chromosomes. Both extra chromosomes were stained with a chromosome 9 painting probe derived from chromosome 9 (Fig. 1D1, 2). The chromosome 9 short arm and long arm subtelomere probes showed that the extra chromosomes had 9p ends at both ends (Fig. 1D1’, 2’). With the chromosome 9 centromere probe, the extra chromosome in 47,XX cells had one stained centromere. However, in 48,XX cells, one extra chromosome had one stained centromere, and another extra chromosome had two stained centromeres (Fig. 1D 1” and 2”). The combined G-band and FISH results indicated 9p hexasomy/tetrasomy mosaicism (47,XX,+der(9)t(9;9)(p24;q13)/48,XX, +der(9)t(9;9)(p24;q13),+idic(9)(q13)). Microarray chromosomal examination was performed to evaluate the gain regions. The results revealed a 9p gain more distal to 9p24, and it was suggested that the 9p24 region constitutes hexasomy at position 48,XX. These results were consistent with the diagnosis of 9p hexasomy/tetrasomy (Fig. 1D, E).

VPS and endoscopic coagulation (EC) were selected as treatments after the patient was admitted to our hospital. Based on previous reports, poor CSF control is expected when only VPS is performed. After surgery, the patient developed significant ascites accumulation, requiring the placement of an abdominal drain. A large amount of ascitic fluid was drained from the abdomen, which resulted in the subsequent development of renal failure due to dehydration. During the course of the patient’s illness, she experienced symptomatic seizures and hemorrhagic stroke.

Although her renal function improved with high-volume fluid replacement, peritoneal drainage was still necessary. Therefore, the right choroid plexus was resected. Postoperatively, her CSF excretion temporarily decreased but subsequently increased rapidly. During the procedure, hyponatremia and seizures due to loss of CSF were observed. CSF excretion from the drain remained high. Therefore, left choroid plexus resection was performed to further reduce CSF excretion. Thereafter, the CSF excretion decreased, the fluid balance stabilized without intracranial hypertension or ascites, and the patient was discharged. After discharge, her gross motor function gradually improved.

Pathological examination of the excised choroid plexus revealed no dysplasia and 1–2% positivity for MIB-1, a well-known proliferation marker for the evaluation of proliferating cells. The patient was diagnosed with diffuse villous hyperplasia of the choroid plexus.

A comparison of this patient with previous patients with 9p gain complicated by CPH suggested that the severity of the disease in this patient was greater than that reported previously (Table 1)^1–7^. Ten cases have been reported in the past, including five cases of trisomy, one case of trisomy/disomy mosaic, and four cases of tetrasomy. VPS was performed as the initial surgical procedure in seven patients, of whom at least five (three patients with trisomy, one patient with trisomy/disomy mosaic, and one patient with tetrasomy) required additional surgical treatment because of the presence of large amounts of ascites or other causes. EC, ventriculoatrial shunt (VAS), and endoscopic third ventriculostomy were chosen as additional surgical treatments.

**Table 1:** VPS ventriculoperitoneal shunt, EC endoscopic coagulation, VAS ventriculoatrial shunt, ETV endoscopic third ventriculostomy.

No	sex	First surgical procedure (age)	Additional surgical procedure (age)	9p gains	reference
1	M	VPS (6 m)	none	tetrasomy/disomy mosaic	^1^
2	F	VPS (11 m)	none	trisomy	^1^
3	F	VPS (1 y)	EC (3 y)	tetrasomy^a^	^2^
4	M	VPS (10w)	VAS (8 y)	trisomy	^3^
5	F^b^	VPS (2 y)	VAS (2 y) EC, VPS (16 y)	trisomy	^3,4^
6	F^b^	none	-	trisomy	^3^
7	F	none	-	tetrasomy/disomy mosaic	^3^
8	M	none	-	tetrasomy/disomy mosaic	^5^
9	F	VPS (14 m)	ETV, EC, anterior choroidal artery embolization (14 m)	trisomy	^6^
10	M	VPS (6 m)	EC, VAS (15 m)	trisomy/disomy mosaic	^7^
11	F	VPS, EC (10 m)	bilateral choroid plexus resection (10 m)	hexasomy/tetrasomy mosaic	Present case

^a^mosaicism is not described.

^b^twins.

In comparison with the findings of previous reports, in this case, additional surgical procedures were required relatively early. In addition, additional surgical treatment was required for this patient despite the initial treatment with VPS and EC to reduce CSF production. Additional surgical treatment involved resection of the bilateral choroid plexus. Although choroid plexus resection is performed for choroid plexus tumors and CPH without chromosomal disorders, no cases of CPH associated with 9p gain requiring choroid plexus resection have been reported. In the treatment strategy, it was expected that an additional shunt, such as a VAS, would be insufficient to reduce the patient’s CSF leakage; thus, choroid plexus resection was selected.

The need for choroid plexus resection in this patient may be attributed to the greater number of 9p gains in this patient than in previous patients. Although identifying the causative gene is difficult, it is suggested that a gene region on 9p will have a quantitative effect on choroid plexus hyperplasia. If 9p gain is associated with hydrocephalus and if there is a high copy number of 9p, treatments to reduce CSF production, such as choroid plexus resection, should be considered.

## Supplementary information

Supplementary information

Supplementary information legends
