# Supplementary figures and images for: Bilateral choroid plexus resection in a 9p hexasomy/tetrasomy mosaic patient
Source: Hum Genome Var. 2024 Feb 26;11:9. doi: 10.1038/s41439-024-00268-x (PMC10897453; doi:10.1038/s41439-024-00268-x)

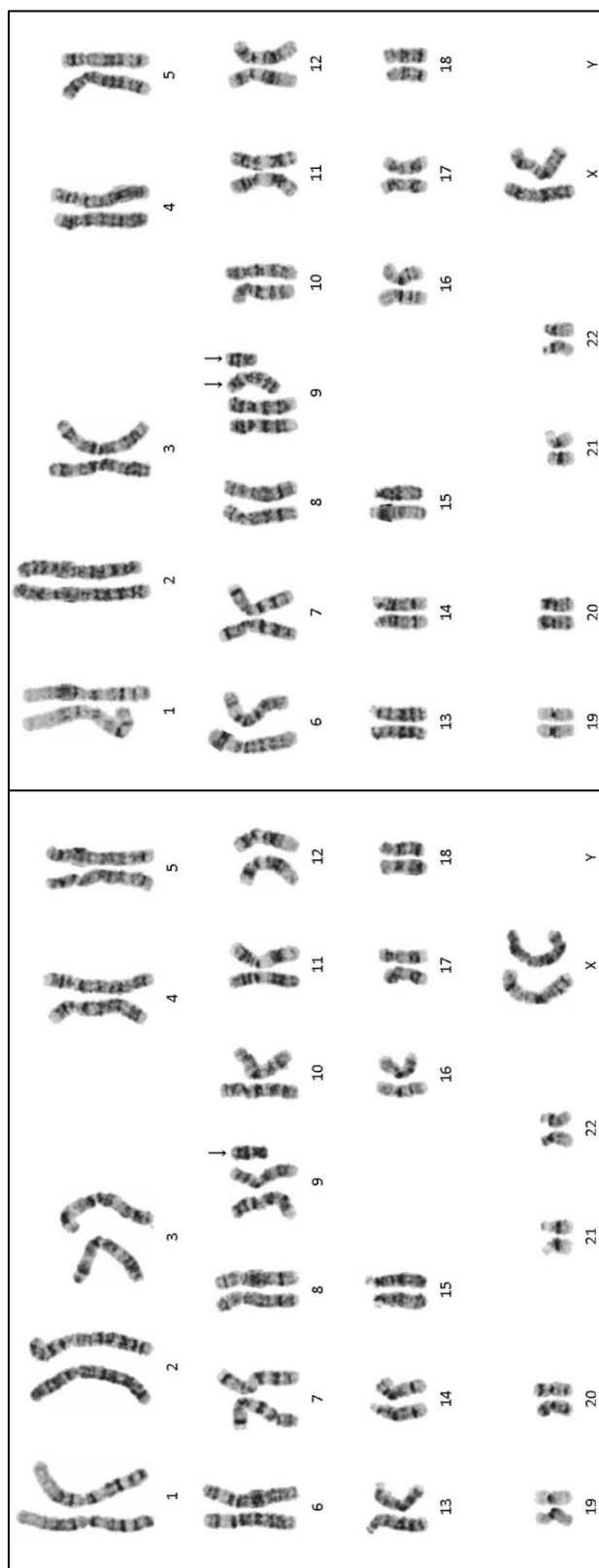

Supplement: Supplementary file 1 — Supplementary information [file 41439_2024_268_MOESM1_ESM.pdf]
