# Supplementary material for: Bilateral choroid plexus resection in a 9p hexasomy/tetrasomy mosaic patient
Source: Hum Genome Var. 2024 Feb 26;11:9. doi: 10.1038/s41439-024-00268-x (PMC10897453; doi:10.1038/s41439-024-00268-x)
Supplement: Supplementary file 2 — Supplementary information legends [file 41439_2024_268_MOESM2_ESM.docx]

**Supplementary Information legends**

Results for the G-band. The mosaic of chromosomes 47,XX and 48,XX was obtained from 10 cells each.
